# Effects of protein intake prior to carbohydrate-restricted endurance exercise: a randomized crossover trial

**DOI:** 10.1186/s12970-020-0338-z

**Published:** 2020-01-28

**Authors:** Mads S. Larsen, Lars Holm, Mads V. Svart, Astrid J. Hjelholt, Mads B. Bengtsen, Ole L. Dollerup, Line B. Dalgaard, Mikkel H. Vendelbo, Gerrit van Hall, Niels Møller, Ulla R. Mikkelsen, Mette Hansen

**Affiliations:** 1 Department of Public Health, Aarhus University, Dalgas Ave. 4, 8000 Aarhus C, Denmark; 2 Arla Foods Ingredients Group P/S, Viby J, 8260 Denmark; 3 School of Sport, Exercise and Rehabilitation Sciences, University of Birmingham, Birmingham, UK; 4 Medical Research Laboratory, Institute for Clinical Medicine, Aarhus University, Aarhus C, Denmark; 5 Department of Endocrinology, Aarhus University Hospital, Aarhus N, Denmark; 6 Department of Biomedicine, Aarhus University, Aarhus C, Denmark; 7 Department of Nuclear Medicine and PET-Centre, Aarhus University Hospital, Aarhus N, Denmark; 8 Clinical Metabolomics Core Facility, Clinical Biochemistry, Rigshospitalet, Copenhagen, Denmark; 9 Department of Biomedical Sciences, Faculty of Health and Medical Sciences, University of Copenhagen, Copenhagen, Denmark

**Keywords:** Carbohydrate restriction, Dietary protein, Endurance training, Protein metabolism

## Abstract

**Background:**

Deliberately training with reduced carbohydrate availability, a paradigm coined *training low*, has shown to promote adaptations associated with improved aerobic capacity. In this context researchers have proposed that protein may be ingested prior to training as a means to enhance the protein balance during exercise without spoiling the effect of the low carbohydrate availability. Accordingly, this is being practiced by world class athletes. However, the effect of protein intake on muscle protein metabolism during *training low* has not been studied.

This study aimed to examine if protein intake prior to exercise with reduced carbohydrate stores benefits muscle protein metabolism in exercising and non-exercising muscles.

**Methods:**

Nine well-trained subjects completed two trials in random order both of which included a high-intensity interval ergometer bike ride (day 1), a morning (day 2) steady state ride (90 min at 65% VO_2_peak, 90ss), and a 4-h recovery period. An experimental beverage was consumed before 90ss and contained either 0.5 g whey protein hydrolysate [WPH]/ kg lean body mass or flavored water [PLA]. A stable isotope infusion (L-[*ring*-^13^C_6_]-phenylalanine) combined with arterial-venous blood sampling, and plasma flow rate measurements were used to determine forearm protein turnover. Myofibrillar protein synthesis was determined from stable isotope incorporation into the vastus lateralis.

**Results:**

Forearm protein net balance was not different from zero during 90ss exercise (nmol/100 ml/min, PLA: 0.5 ± 2.6; WPH: 1.8, ± 3.3) but negative during the 4 h recovery (nmol/100 ml/min, PLA: − 9.7 ± 4.6; WPH: − 8.7 ± 6.5); no interaction (*P* = 0.5) or main effect of beverage (*P* = 0.11) was observed. Vastus lateralis myofibrillar protein synthesis rates were increased during 90ss exercise (+ 0.02 ± 0.02%/h) and recovery (+ 0.02 ± 0.02%/h); no interaction (*P* = 0.3) or main effect of beverage (*P* = 0.3) was observed.

**Conclusion:**

We conclude that protein ingestion prior to endurance exercise in the energy- and carbohydrate-restricted state does not increase myofibrillar protein synthesis or improve net protein balance in the exercising and non-exercising muscles, respectively, during and in the hours after exercise compared to ingestion of a non-caloric control.

**Trial registration:**

clinicaltrials.gov, NCT01320449. Registered 10 May 2017 – Retrospectively registered, https://clinicaltrials.gov/ct2/show/NCT03147001

## Introduction

During the past decade, ‘periodization’ has been a hot topic in sports nutrition [1, 2]. The term ‘periodized nutrition’ refers to strategic manipulation of nutrient availability during training to promote specific adaptations. Among the various strategies, undertaking training with low carbohydrate availability has received much attention by researchers [2–4]. This practice is popularly referred to as *training low*. Several studies have shown that commencing endurance-type exercise with low endogenous and exogenous carbohydrate availability 1) augments the mobilization of lipids for oxidation and 2) enhances activation and gene transcription encoding key proteins underpinning adaptations associated with a phenotype of improved endurance capacity [5–10]. Among these proteins, AMP-activated kinase (AMPK) and peroxisome proliferator-activated receptor gamma coactivator (PGC)-1α are reputed to play particularly important roles. AMPK acts as a myocellular fuel gauge promoting fatty acid oxidation, while PGC-1α is considered a master regulator of mitochondrial biogenesis [3]. Congruently, *training low* is employed by athletes to achieve greater aerobic and fat oxidation capacity [11–13].

Nevertheless, *training low* comes with a caveat: Typically, amino acids contribute ~ 5% of the energy cost during endurance exercise [14]. However, commencing training with low carbohydrate availability may double this contribution, seemingly brought about by accelerating muscle protein breakdown [15–17]. Indeed, low endogenous carbohydrate availability augments leucine oxidation [16] and amino acid release from the working muscle [16, 17] and attenuates protein synthesis during exercise [16]. Thus, repetitive practice over an extended period has raised concerns among scientists [11, 18, 19], as it may affect skeletal muscle mass negatively, and possibly compromise athletic performance. Accordingly, a recent study estimated that commencing a 10-km run with reduced carbohydrate availability increases daily protein requirement by 0.12 g/kg.

As a means to ameliorate the augmented muscle protein breakdown, a few studies have examined the effect of protein feeding before and during *training low* [20, 21]. These studies have shown that protein ingestion before training in a glycogen-depleted state does not seem to compromise lipolysis and fat oxidation compared to non-caloric placebo treatment [20, 21]. Furthermore, Taylor et al. demonstrated that protein intake before glycogen-depleted exercise did not influence acute AMPK phosphorylation and PGC-1α mRNA transcription. The authors also suggest that the protein provision before and during the glycogen-depleted training bout appeared to induce greater dephosphorylation (i.e. activation) of the eukaryote elongation factor 2 (eEF2) compared to the placebo trial [20]. Because of its role as a molecular regulator of elongation, the authors inferred that protein ingestion during *training low* reduced suppression of muscle protein synthesis during exercise [20]. As acknowledged by the authors, a critical limitation to their study is the lack of direct assessment of muscle protein turnover.

Regardless of the limited scientific evidence, world-class endurance athletes evidently already practice protein ingestion before and/or during *training low* sessions [12, 13].

Intrigued by the results by Taylor et al. [20] and the reputed practice of world class athletes, we aimed to conduct a proof-of-principle trial to determine how a protein bolus ingested prior to commencing endurance training in a carbohydrate/energy-restricted condition affects skeletal muscle protein metabolism. We examined muscle protein synthesis in the highly active muscle vastus lateralis and protein kinetics (i.e., net balance, synthesis and breakdown) in the forearm to represent non-exercising muscle tissue. The reason for this choice was that whole-body protein kinetics may not reflect that of the skeletal muscle tissue.

We hypothesized that protein ingestion prior to *training low* would stimulate muscle protein synthesis in the exercising muscles and would improve protein net balance in non-exercising muscle. Additionally, we examined cell signaling and gene expression associated with myofibrillar and mitochondrial biogenesis to assess how these parameters were affected by protein ingestion prior to *training low*.

## Methods

### Subjects

Twelve competitive male triathletes or cyclists were included in the study after being deemed healthy and fit for participation. The inclusion criteria required subjects to be healthy (no metabolic abnormalities), non-smokers, 18–50 years old with a relative V̇O_2peak_ above 55 ml O_2_/kg/min, and a history of five or more training sessions per week for 6 months prior to inclusion. All participants were given oral and written information and gave their written consent to participate. The study was approved by the local ethical committee of the Central Denmark Region (M-20110035) and was conducted in accordance with the Declaration of Helsinki. The study was registered with clinicaltrials.gov (NCT01320449).

### Pretesting

Prior to the experiment, a routine blood sample was collected to determine the subject’s general metabolic health. The blood samples were collected in the fasted state and analyzed for levels of low density lipoprotein, high density lipoprotein, triglycerides, leucocytes, aminotransferase, aspartate aminotransferase, alkaline phosphatase, albumin, testosterone, thyroid stimulating hormones, triiodothyronine, creatine kinase, c-reactive protein and hemoglobin to get an indication of any undiagnosed conditions that the participant might unknowingly have suffered from.

Body composition was determined using dual-energy x-ray absorptiometry (DXA; GE Lunar DXA scan, GE Healthcare, USA).

Peak power output (PPO) and peak oxygen consumption (V̇O_2peak_) were determined independently of the experimental trials and DXA scan, using an incremental test-to-exhaustion on an electronically braked bicycle ergometer (SRM, Julich, Germany) as described previously [22]. V̇O_2peak_ was defined as the highest oxygen uptake attained during any 30 s of the test. Respiration was analyzed using an Oxigraf O2CPX (Oxigraf O2CPX, Model Part Number 07–0464, Oxigraf Inc) with Innocor 8.00 software (Innovision ApS, Odense, Denmark). PPO was calculated from the highest completed stage of exercise intensity plus the fraction of time spent in the final uncompleted stage [22]. The individual PPO was used to determine the prescribed intensities during the experimental trials.

### Study design

**Fig. 1:**
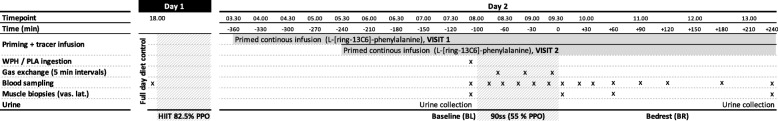
Overview of study design. On day 1, all meals were provided. At 1900 subjects commenced 10 × 5 min intervals at 82.5% (HIIT) of individual peak power output (PPO) on a customized ergometer bike. L-[ring-^13^C_6_-phenylalanine] was initiated during the night. Upon awakening (day 2), blood, muscle and urine samples were collected before commencement of a 90 min steady state ride (55% PPO). Subsequently subjects rested in a supine position for 4 h. Samples were collected as indicated

The protocol is visualized in Fig. 1. In a single-blinded counterbalanced crossover trial, subjects were studied on two occasions (Visit 1 and Visit 2) separated by a minimum of 14 days. The contents of the experimental beverage (whey protein hydrolysate [WPH] or placebo [PLA]) differed; otherwise, the experiments were conducted in a similar manner. Each experimental period covered 2 days and consisted of two exercise sessions performed on an ergometer bike, similar to the protocol described by Lane et al. [7]: the first high-intensity interval training (HIIT) session was performed in the evening of Day 1. The second session, a 90 min steady state (90ss) cycling bout, was performed at 08.00 h on the following morning (Day 2). All foods consumed on Day 1 were standardized and matched between the two visits. Experimental beverages were ingested immediately prior to 90ss. Ingestion of food and beverages other than water and the experimental beverage was not allowed from arrival at the laboratory until completion of Day 2. Throughout Day 2, fractional protein synthesis rates (FSR) and muscle protein turnover were determined using stable isotope tracer techniques.

### Experimental days

At both visits, the subjects reported to the laboratory at 1800 h on Day 1. After voiding their bladder, they commenced the HIIT session. Afterwards a catheter was placed in an antecubital vein. A background blood sample was collected and the catheter was kept patent by continuous infusion of 9% saline until 0330 h (0530 h at the second visit), when a primed (6.0 μmol/kg lean body mass [LBM]) continuous (6.0 μmol/kg LBM/h) infusion of L-[*ring*-^13^C_6_]-phenylalanine (Cambridge Isotopes, Andover, MA, USA) was initiated and maintained until the end of Day 2 (1330 h). Tracer solutions were sterilely prepared and tested free of bacteria and pyrogens before use. In the morning of Day 2, a catheter was placed contralaterally to the tracer infusion in an antecubital vein, in a retrograde direction, for deep venous sampling. Another catheter was placed in a dorsal hand vein. The hand was heated to ~ 55 °C for sampling of arterialized blood. Venous occlusion plethysmography was used to determine forearm blood flow.

Immediately before commencing 90ss, a baseline muscle biopsy and blood samples were obtained, and the experimental beverage was consumed. After completion of 90ss, the subjects were kept in a supine position for the rest of the experiment. A muscle biopsy was obtained immediately, and at 1 and 4 h of after termination of the 90ss. Urine was collected in two intervals. Batch 1: From the initiation of HIIT to the commencement of 90ss. Batch 2: From the commencement of 90ss to the end of the study period.

### HIIT

After a self-chosen 10-min warm up, the HIIT session was performed on an ergometer bike (LC4, Monark AB, Vangsbro Sweden). The HIIT consisted of ten 5-min bouts at ~ 82.5% of individual PPO with 90 s of active recovery between the intervals. This protocol is a modified version of one described by Stepto et al., which showed a ~ 50% reduction in muscle glycogen levels [7, 23].

### 90ss

The work rate was fixed at 55% of PPO. The bicycle ergometer was modified by addition of customized aerobars with an attached table for hand placement (subjects did not grip the handle bars). The subjects were instructed to remain positioned in the aerobars throughout the exercise bout to minimize muscle activity in the forearms while cycling. VO_2_ was measured during 5-min intervals every 30 min of exercise. Ratings of perceived exertion were recorded prior to VO_2_ measurements. Heart rate was measured continuously throughout 90ss. The work performed during the HIIT and 90ss sessions at Visit 1 was replicated at Visit 2.

### Diet and exercise control

On Day 1, a standardized portion-controlled diet containing (per kg body weight) 6.8 g carbohydrate, 1.8 g protein, and 1.7 g fat was provided for all participants. No exercise, alcohol, caffeinated drinks, or medical drugs were allowed in the 24 h prior to HIIT. Water was allowed ad libitum at all times during the experimental periods.

### Experimental beverages

were provided immediately before starting the 90ss session and contained either 0.5 g/kg LBM WPH (Lacprodan® HYDRO.365, Arla Foods Ingredients Group P/S, Viby J, DK) with 10% L-[*ring*-^13^C_6_]-phenylalanine enrichment (Table 1) or a non-caloric placebo matched for flavor. The amino acid content of the protein beverage is appended (see Additional file 1).

**Table 1:** Protein beverage content / 100 g

Water	85.52 g
Whey protein	10.00 g
Carbohydrate	1.33 g
Fat	0.03 g
Energy	45.6 kcal

All subjects ingested a drink with a protein content equivalent to 0.5 g/kg LBM

### Blood sampling and analysis

All blood samples were collected into coated vacuum blood collection tubes. Plasma amino acid concentrations and enrichments were determined as described previously [24]. Concentrations of insulin, cortisol, serum glucose, and plasma-free fatty acids (FFA) were quantified as described previously [25, 26]. Hemoglobin (HemoCue Hb 201^+^, Ängelholm, Sweden) and 3-hydroxybuterate (FreeStyle Precision; Abbott Diabetes Care) were measured immediately after sampling. Analyses of plasma and urinary urea and plasma ammonium were performed using absorption photometry (Cobas 6000, Roche, Basel, CH and Chemistry XPT System, Simens Healthcare A/S, Ballerup, DK).

### Muscle biopsies sampling and analyses

The muscle biopsies (~ 250 mg) were obtained from the vastus lateralis muscle under local anesthesia (10 ml Xylocain® 10 mg/ml, AstraZeneca, Sweden) using a 5 mm Bergström needle with manual suction. At each visit, all samples were obtained from the same randomly chosen leg (dominant or non-dominant) through separate incisions. After removing visible blood, fat, and connective tissue, the samples were snap frozen and stored at − 80 °C until further analysis.

#### Stable isotope analysis

For practical reasons, a single biopsy approach was used to assess basal muscle protein FSR at Visit 1 [24, 27, 28]. This value represents basal FSR for both treatments in the statistical analysis. Myofibrillar and mitochondrial proteins were isolated as described previously [29]. Briefly, each muscle sample (~ 25 mg wet weight) was homogenized in 1 ml homogenization buffer (0.02 M Tris [pH 7.4], 0.15 M NaCl, 2 mM EDTA, 0.5%, TritonX-100 and 0.25 M sucrose) and spun at 800 g at 5 °C. This procedure was repeated. The resulting supernatants were combined and spun at 10,000 g (5 °C), washed, and spun in 1 ml 70% ethanol (1600 g, 5 °C) to pellet the mitochondrial proteins. The original pellet containing myofibrillar and connective tissue proteins was vortexed, left overnight (5 °C) in a KCl buffer (0.7 M KCl, 0.1 M pyrophosphate) and then spun at 1600 g (20 min, 5 °C). The supernatant was discarded and 99% ethanol was added and left for 2 h. This procedure was repeated. The resulting myofibrillar protein pellet was hydrolyzed at 110 °C in 6 M HCl overnight. Both the mitochondrial and myofibrillar amino acids were purified over prepared resin columns (AG 50 W-X8 resin; Bio-Rad Laboratories, Hercules, CA), eluted with 4 M NH_4_OH, and evaporated under a stream of nitrogen before being derivatized as the N-acetyl-propyl derivative as described elsewhere [30]. Unfortunately, several samples from the mitochondrial fraction resulted in signals too low to detect on the GC/C/IRMS. Therefore, we were unable determine the FSRs for mitochondrial protein.

#### Calculations

Calculations of forearm phenylalanine kinetics and muscle protein FSRs were performed as described by Smith et al. [31] and Holm et al. [24], respectively. Calculations are appended (see Additional file 2).

#### Gene expression

Approximately 20 mg of muscle tissue was homogenized using a Precellys 24 Tissue Homogenizer (Bertin Instruments, Rockville, MD, USA). Total RNA was isolated using an RNA KIT (Qiagen, #217004) according to the manufacturer’s instructions. Concentrations and purity were confirmed by spectroscopy. All reagents and equipment used were from Applied Biosystems™ (Foster City, Ca, USA). Five hundred nanograms of RNA was converted to cDNA using a High-Capacity cDNA Reverse Transcription Kit, and real time PCR was performed with Fast Advanced Mastermix using a StepOnePlus. Genes (targets IDs) were: *CPT1B* (HS03046298 s1), *COX4I1* (HS00971639 m1), *PPARGC1* (HS00173304 m1), *TFAM* (HS01082775 m1), *RPLP0* (HS99999902 m1) and *GAPDH* (HS99999905 m1). Data were converted to fold changes from PRE using the Delta Delta Ct method [32] with RPLP0 as the internal control. Ct values obtained for *RPLP0* were not affected by treatment or time.

#### Western blots

Approximately 30 mg of muscle tissue was used to determine protein concentrations via Bradford assays (Bio-Rad, Ca, USA). Target proteins were resolved by 4–15% sodium dodecyl sulfate–polyacrylamide gel electrophoresis (Criterion TGX gradient 4–15%, Bio-Rad) with three molecular markers (Precision Plus All Blue, Bio-Rad) and an internal control before being electroblotted to polyvinylidene difluoride membranes (Bio-Rad, CA, USA). The membranes were blocked in I-block™ Protein-based Blocking Reagent (Applied Biosystems™, Bedford, MA, USA) and incubated overnight in primary antibodies followed by TBS-T wash and incubation (1 h) with secondary antibodies. Primary antibodies (cat. no.): p-mTORser2448 (2971), p-p38MAPKthr180/tyr182 (4511), p-p70S6Kthr389 (9205), p-eIF4Eser209 (9741) and p-p53ser15 (9284), all from Cell Signaling Technology (Danvers, MA, USA).

Horseradish peroxidase-conjugated secondary antibodies: Goat anti-rabbit IgG H&L (ab6721; Abcam, Cambridge, UK) and Anti-mouse IgG (7076; Cell Signaling Technology (Danvers, MA, USA).

Proteins were visualized using a chemiluminescence detection system (Thermo Scientific, IL, US) and quantified using a Bio spectrum 500 Imaging System (UVP, Cambridge, UK). All Blue Standards (Bio-Rad, CA, USA) were used as molecular weight markers.

### Statistical analysis

An a priori sample size of ten subjects was established based on previous tracer kinetics data from trials similar to ours [33, 34]. For each treatment (PLA or WPH), time-weighted arithmetic means were calculated for each time period (BL, 90ss and BR) to determine a conjoined and clinically meaningful response for plasma metabolites, hormones and tracer kinetic data. Data on muscle signaling were analyzed and expressed as relative fold change from baseline corresponding to the biopsy obtained immediately before commencing 90ss at each visit. For gene expression, delta CT values were analyzed statistically but expressed as relative fold changes from baseline. Statistical analyses were conducted using a repeated-measures mixed effects model. Treatment, time period (BL, 90ss and BR), and order-of-treatment were included as independent variables. Subject and visit (1 or 2) were included in the random part of the model to account for any carry-over effect and random intercepts for the subjects, respectively. This was followed by a joint test of two-way interactions. Significance was set at an α-level of < 0.05. If *F*-ratios were significant, Bonferroni post hoc tests were applied to locate differences. Normality and heteroskedasticity of all data were checked by visually inspecting QQ-plots and plots of residuals versus the fitted values. No obvious deviations of normality were detected. Data that were not homoscedastic (3-hydroxybutyrate, plasma ammonia and amino acid concentrations) were log-transformed for the statistical analyses but not presented as such. Treatment differences in urinary nitrogen excretion and volume of voiding were analyzed using paired t-tests. Values are presented as means ± standard deviation (SD). All statistical analyses were performed using STATA version 14.2 (StataCorp LP, Collage Station, TX, USA).

## Results

### Study participants

Of the 12 subjects included in the study, two failed to attend the experimental visits; one due to injury unrelated to the study and one due to scheduling difficulties. One subject did not attend the second trial for personal reasons and was therefore excluded from all analyses. Subject characteristics are shown in Table 2.

**Table 2:** Subject characteristics (*n* = 9)

Age (years)	28.6 ± 9.0
Body mass (kg)	73.1 ± 4.5
DXA body mass (kg)	73.7 ± 4.3
Lean body mass (kg)	60.1 ± 4.2
VO_2_peak (ml O_2_ • kg^− 1^ • min^− 1^)	60.0 ± 2.6
Peak power output (watt)	364 ± 24

Data are mean ± SD

### HIIT and 90ss

The mean PPO attained during pretesting was 364 ± 24 watts. The average power output across intervals was 281 ± 21 watts. On average, participants dropped ~ 4.6% between interval one and ten (95% CI: − 6.7, − 2.4; *P* < 0.001). Concomitantly, the rating of perceived exertion rose from ~ 14 to ~ 18 on the Borg scale (*P* < 0.001). The 90ss sessions were performed at an average power of 55.6 ± 2.6% (PLA) and 54.9 ± 2.9% (WPH) of PPO. During both trials, the mean respiratory exchange ratio dropped from 0.87 to 0.84 throughout the 90ss session (*P* < 0.001).

### Blood profile

**Fig. 2:**
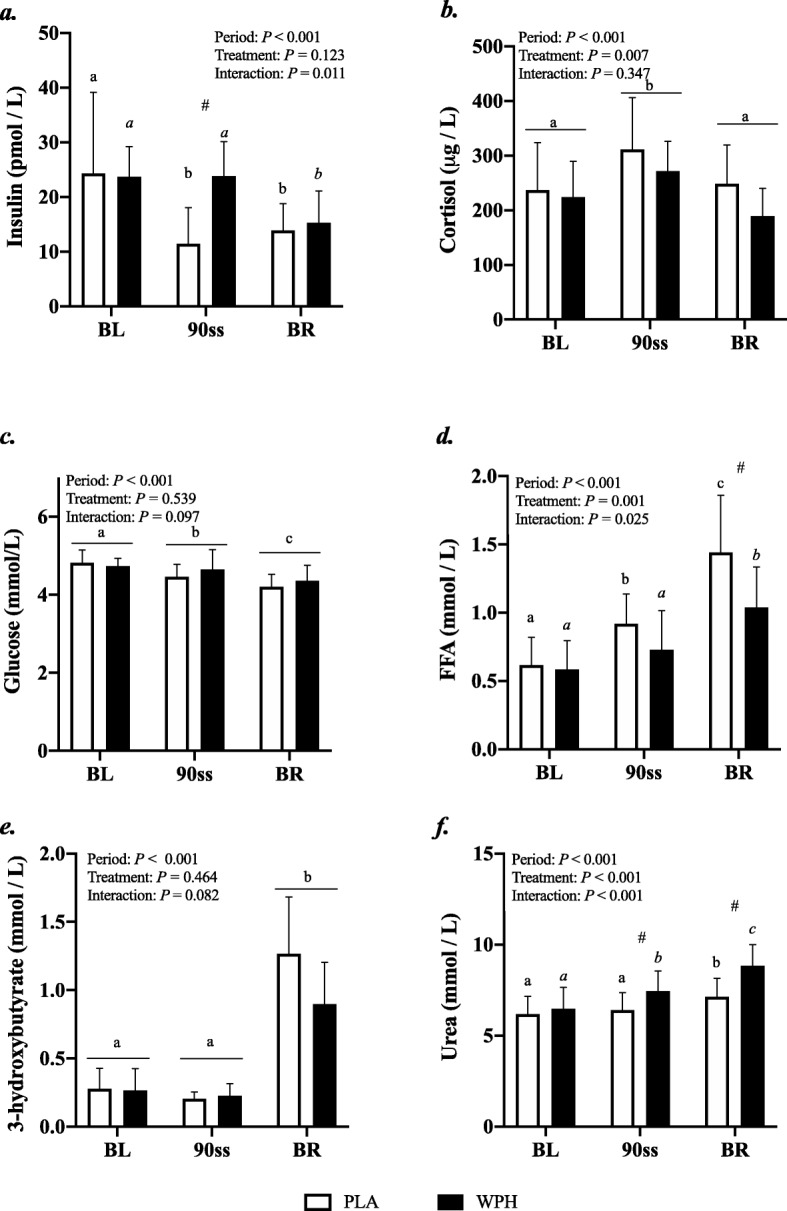
Blood parameters. Change in hormone and metabolite levels during BL, 90ss and BR. Insulin (**a**), cortisol (**b**), glucose (**c**), free fatty acids (FFA) (**d**), 3-hydroxybutyrate (**e**), urea (**f**). Data are shown as means ± SD (n = 9); *P* < 0.05. Means within each trial with different subscripts are significantly different from each other; WPH subscripts are in *cursive*. ^#^ Significant difference between PLA and WPH at each respective timepoint

**Fig. 3:**
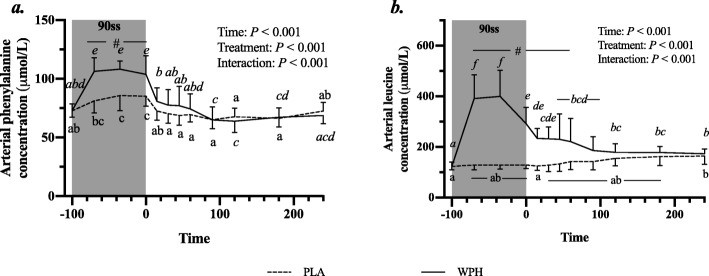
Arterial concentrations of phenylalanine (**a**) and leucine (**b**) at baseline (BL), during 90 steady state exercise (90ss) and during bed rest recovery (BR) with PLA or WPH ingestion. Values are means ± SD (*n* = 9); *P* < 0.05. Means within each trial with different subscripts are significantly different from each other; WPH subscripts are in *cursive*. ^#^ Significant difference between PLA and WPH at each respective timepoint

**Fig. 4:**
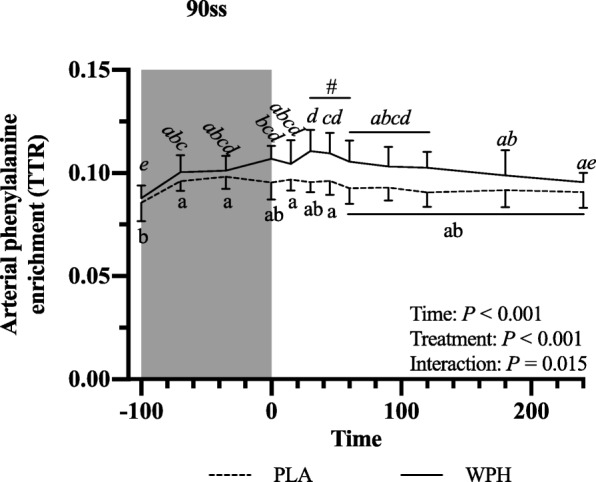
Arterial phenylalanine enrichment at baseline (BL), during 90 steady state exercise (90ss) and bed rest recovery (BR) with PLA or WPH ingestion. Values are means ± SD (*n* = 9); *P* < 0.05. Means within each trial with different subscripts are significantly different from each other; WPH subscripts are in *cursive*. ^#^ Significant difference between PLA and WPH at each respective timepoint

All blood profiles are illustrated in Fig. 2. Serum insulin (Fig. 2a) was 12.5 pmol/L higher in WPH than in PLA during 90ss (*P* = 0.02; 95% CI: 0.9, 24.1), as insulin levels in PLA dropped 12.3 pmol/L from BL levels (*P* = 0.03; 95% CI: − 23.9, − 0.7). Both treatments showed lower insulin levels during BR than at BL (*P* < 0.05; 95% CI: − 10.1, 13.1). Plasma cortisol increased during 90ss for both treatments and returned to BL levels during BR. No time-treatment interaction was observed (*P* = 0.34); yet, statistical analysis showed higher plasma cortisol throughout PLA compared with WPH (+ 36.1 μg/L; *P* < 0.01; 95% CI: 62.4, 9.8; Fig. 2b). Blood glucose was reduced throughout Day 2 in both WPH and PLA (Fig. 2c; *P* < 0.001). Though not statistically significant, there tended to be an interaction with WPH showing higher glucose levels than PLA (*P* = 0.097). FFA concentrations in PLA were increased during 90ss (0.92 ± 0.22 mmol/L; *P* = 0.03; 95% CI: 0.02, 0.58; Fig. 2d) compared with BL (0.62 ± 0.20 mmol/L) and further during BR (1.44 ± 0.10 mmol/L; *P* < 0.001). In WPH, the FFA levels were unchanged from BL during 90ss (mmol/L: 0.59 ± 0.21 vs 0.73 ± 0.29; *P* = 1.0; 95% CI: − 0.14, 0.43), but rose during BR (1.04 ± 0.30 mmol/L; *P* < 0.001; 95% CI: − 0.17, 0.73). Plasma 3-hydroxybutyrate concentrations were unchanged during 90ss for both treatments (Fig. 2e; *P* > 0.05) but were increased for both treatments during BR (*P* < 0.001). There tended to be a period x treatment interaction (*P* = 0.082). Plasma urea rose throughout the experimental day in both treatments (Fig. 2f; *P* < 0.001) but was higher for WPH (+ 1.0 mmol/L; 95% CI: 0.5, 1.6; *P* < 0.001) than for PLA during 90ss and BR (+ 1.6 mmol/L; *P* < 0.001; 95% CI: 1.1, 2.2). Plasma ammonium concentrations were increased during 90ss (μmol/L: PLA, 85.8 ± 21.6; WPH, 87.7 ± 22.3) for both treatments (*P* < 0.001) and returned to BL (μmol/L: PLA, 44.7 ± 9.6; WPH, 44.8 ± 9.5) during BR (μmol/L: PLA, 38.8 ± 7.3; WPH, 38.6 ± 4.8). Arterial phenylalanine concentration increased during 90ss in both treatments, but more so in WPH than in PLA (Fig. 3a; *P* < 0.001). Arterial leucine concentrations increased after protein consumption only and stayed elevated into BR (Fig. 3b; *P* < 0.001). Arterial phenylalanine enrichment had reached a plateau before commencing 90ss. A slight increase was observed immediately after 90ss in WPH (Fig. 4; *P* < 0.001).

Urinary urea nitrogen excretion was higher (+ 147 mg/hr.; *P* = 0.03; 95% CI: 13, 281) in WPH (613 ± 165 mg/hr) than in PLA (466 ± 93 mg/hr) during the experimental day (Day 2). Urine production was similar in both treatments (mL/h: PLA, 48 ± 12; WPH, 51 ± 12; *P* = 0.65).

### Forearm protein kinetics

**Fig. 5:**
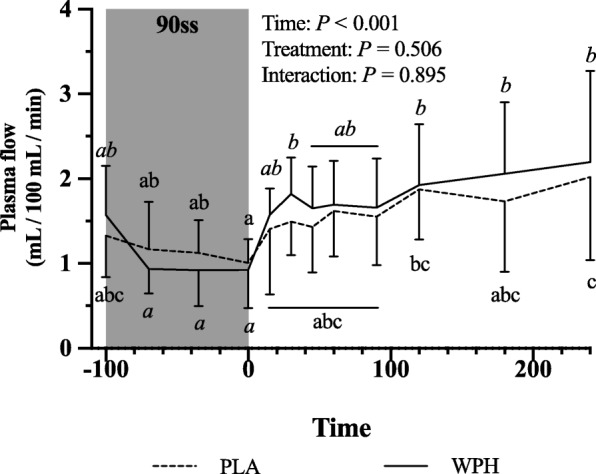
Forearm plasma flow at baseline (BL), during 90 steady state exercise (90ss) and bed rest recovery (BR) with PLA or WPH ingestion. Values are means ± SD (*n* = 9); *P* < 0.05. Means within each trial with different subscripts are significantly different from each other; WPH subscripts are in *cursive*

**Fig. 6:**
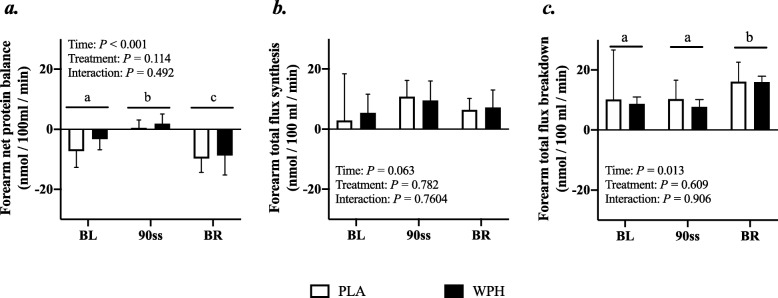
Forearm net protein balance (**a**), forearm protein synthesis (**b**), forearm protein breakdown (**c**) at baseline (BL), during 90 steady state exercise (90ss) and during bed rest recovery (BR) with PLA or WPH ingestion. Values are means ± SD (*n* = 9); *P* < 0.05. Means with different subscripts are significantly different from each other

Forearm phenylalanine net balance was negative at baseline (Fig. 5a). During 90ss, a tendency towards increased rates of amino acid uptake by the forearm (Fig. 5b) was observed. This resulted in a neutral net protein balance in both groups. During BR, the rates of amino acid released from the arm increased (Fig. 5c; *P* = 0.01), yielding a negative net protein balance. During 90ss forearm plasma flow decreased compared to BL (Fig. 6; *P* < 0.001) and increased to a level above BL during BR (Fig. 6; *P* < 0.001).

### Fractional synthetic rate

**Fig. 7:**
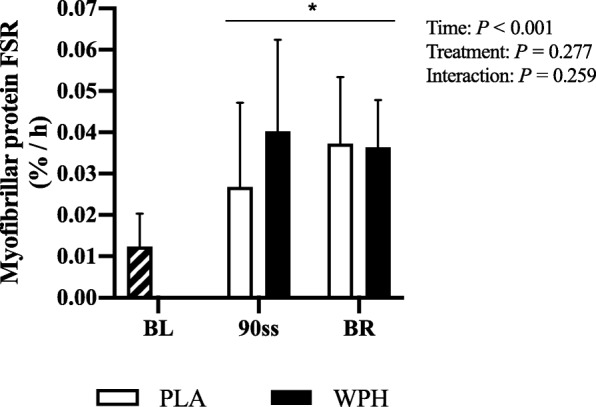
Muscle protein FSR of the m. vastus lateralis during baseline (BL), 90 min steady state exercise (90ss) and bed rest recovery (BR) with PLA or WPH ingestion. BL FSR’s were performed at Visit 1 regardless of treatment (hatched bars). Values are means ± SD (*n* = 9); *P* < 0.05. * Significantly different from BL

Vastus lateralis FSR was increased during 90ss and BR for both treatments (Fig. 7; *P* < 0.05).

### Muscle signalling and gene expression

#### Western blotting

**Fig. 8:**
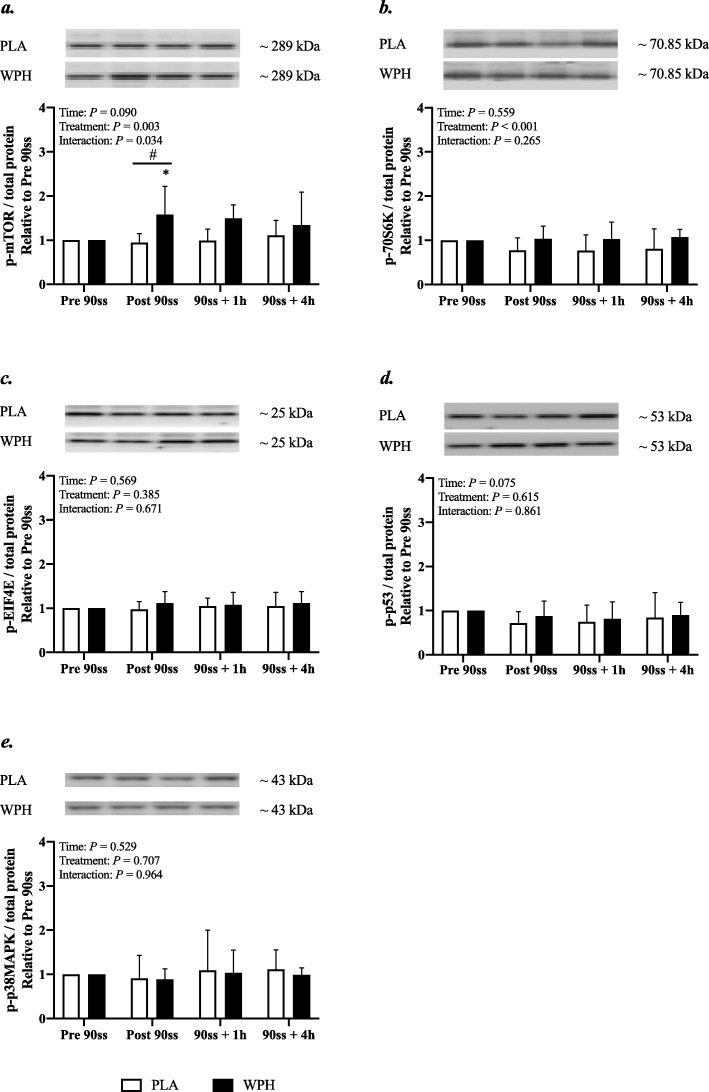
Protein phosphorylation. Mammalian target of rapamycin (mTOR) (**a**), ribosomal protein S6 kinase beta-1 (p70S6K) (**b**), eukaryotic translation initiation factor 4E (EIF4E) (**c**), tumor protein p53 (p53) (**d**), p38 mitogen-activated protein kinases (p38MAPK) (**e**). Western blots representing the time-course effects are presented below the graphs. Based on the applied molecular standards, approximated molecular weights are indicated to the right. *n* = 9 for all timepoints. Values are normalized to PRE 90ss and are expressed as means ± SD; *P* < 0.05. * Significantly different from BL. # Significant difference between trials

In WPH, mTOR phosphorylation was increased immediately after 90ss compared with BL (Fig. 8a; + 58%; 95% CI: 6, 111; *P* = 0.01) and PLA (+ 64%; 95% CI: 6, 121; *P* = 0.017). Also, an overall treatment effect was observed for p70S6K (Fig. 8b; + 20% in WPH; 95% CI: 9, 31; *P* < 0.001). p53 phosphorylation tended (*P* = 0.08) to be decreased immediately (Fig. 8c; − 20; 95% CI: − 38, − 2) and 60 min (− 22%; 95% CI: − 40, − 4) after 90ss across treatments. p-eIF4E and p-p38MAPK were not affected by time or treatment (Fig. 8d & e; *P* > 0.05).

#### Real-time PCR

**Fig. 9:**
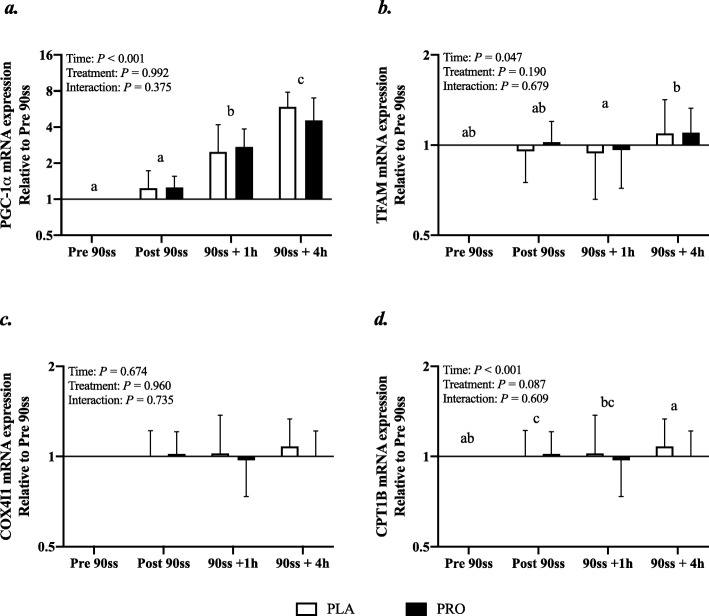
Gene expression. mRNA expression of peroxisome proliferator-activated receptor gamma coactivator 1-alpha (*PGC-1α*) (**a**), mRNA expression of mitochondrial transcription factor A (*TFAM*) (**b**), mRNA expression of cytochrome c oxidase subunit IV (*COXIV*) (**c**), mRNA expression of carnitine palmitoyl transferase 1B (*CPT1B*) (**d**). *n* = 9 for all timepoints. Values are set relative to PRE 90ss and the fold changes are expressed as means ± SD; *P* < 0.05. Means within each trial with different subscripts are significantly different from each other. # Significant difference between trials

In response to 90ss, *PGC1α* mRNA expression increased throughout the day to about fivefold at the end of Day 2 (Fig. 9a; *P* < 0.001). An overall time effect was observed for *TFAM* mRNA, but post hoc tests revealed no differences from baseline for either treatment (Fig. 9b). *CPT1B* was suppressed immediately after 90ss compared with prior to 90ss (Fig. 9c; *P* < 0.001) and there tended to be an overall treatment effect indicating a higher expression of *CPT1B* in the PLA than WPH trial (*P* = 0.09). *COX4I1* was not affected by time or treatment (Fig. 9d; *P* > 0.05).

## Discussion

The main finding of the present study was that supplementation with ~ 35 g of protein (0.5 g / kg LBM) did not enhance protein net balance in the forearm or increase vastus lateralis myofibrillar protein synthesis compared to the placebo treatment, despite inducing a rapid increase in plasma amino acid concentrations that lasted into post-exercise recovery. To our knowledge, this is the first study to examine the direct impact of protein ingestion on muscle protein turnover (*forearm*) and synthesis rates, while training in a state of reduced carbohydrate availability. We used stable isotope tracers to determine protein turnover in the non-exercising muscles (forearm) and fractional protein synthesis in the exercising vastus lateralis muscles during and after bicycle exercise.

Other investigators have hypothesized that protein feeding would mitigate the reputed increase in muscle protein breakdown during exercise by providing substrate for gluconeogenesis and oxidation. A further suggested benefit was that the ingested protein would enhance the muscle protein synthesis rate when training with low exogenous and endogenous carbohydrate availability (*training low*) [20, 35]. Furthermore, previous reports suggest that the enhanced adaptive response induced by *training low* is not hampered by a preceding and/or concomitant protein intake [20, 21]. Indeed, protein ingestion during and/or after endurance type exercise has been shown to enhance muscle protein synthesis [33, 34, 36, 37]. However, our data endorse the notion that endurance exercise blunts the anabolic response to hyperaminoacidemia [33, 37], possibly as a part of a metabolic priority shift towards energy transduction and conservation as proposed by Atherton and Rennie [38].

### Amino acid kinetics and muscle protein synthesis

We observed that vastus lateralis muscle protein synthesis rates were elevated during and after exercise in both the PLA and the WPH trial. Thus, our data confirm the findings of Beelen et al., who also showed an increase in muscle FSR in response to endurance type exercise with and without exogenous protein provision [37]. Based on observed increases in muscle free amino acid concentrations, Beelen and colleagues suggested that the muscle FSR increase may be attributed to an increased provision of endogenously derived amino acids to the working muscle facilitated by increased blood flow. While this seems a plausible explanation, our tracer kinetics data from the forearm does not suggest that less active muscle tissue becomes a supplier of such amino acids given our results show an increased net protein balance from BL to 90ss in both the WPH and PLA trials. Other studies examining combined protein and carbohydrate ingestion during moderately intense endurance exercise have shown enhanced whole-body [34, 37, 39] and leg [33] protein synthesis and net protein balance compared with carbohydrate or non-fed controls. While the inconsistency between these findings and ours may be influenced by the tissue examined, the lack of carbohydrate and/or energy availability in our subjects likely attenuated the anabolic response to hyperaminoacidemia. Furthermore, we cannot dismiss that the provision of exogenous amino acids via the WPH drink may have ameliorated the muscle protein breakdown in the legs, as observed by Hulston et al. (2011) albeit in subjects who were not reduced in muscle glycogen.

Although we did not measure muscle glycogen content directly, we believe that the HIIT session had lowered the muscle glycogen content significantly. Indeed, similar depletion protocols have been shown to reduce muscle glycogen ~ 50% [7, 23]. Furthermore, these studies show that the achieved reductions in muscle glycogen persisted until the following morning [7, 17, 40].

### Blood parameters

In accordance with our tracer kinetics data, showing no effect of protein ingestion, plasma metabolite concentrations indicate that the ingested protein bolus was metabolized rather than used as substrate for protein synthesis in the muscle tissues. Had we had access to intrinsically labelled protein, we could have traced the fate of the ingested amino acids. However, the observations of elevated plasma urea in WPH vs PLA (90ss: + 17%; BR: + 24%), accompanied by a borderline time x treatment interaction for plasma glucose levels, imply that amino acids were used as an energy substrate. Moreover, the WPH treatment yielded an overall lower level of cortisol, suggesting that the more stable blood glucose and larger energy substrate provision rendered the subjects less stressed and possibly less catabolic.

The WPH produced an increase in serum insulin concentration compared with the PLA trial. Even though the plasma insulin levels did not rise above rest levels, the slightly higher insulin levels observed in WPH during 90ss may have borne clinical relevance in suppressing lipolysis [41]. Previous studies have shown that insulin repression during exercise is crucial for FFA mobilization and that failure to suppress insulin during exercise blunts plasma FFA markedly [42, 43]. Accordingly, we observed depressed levels of FFA following exercise in WPH compared to PLA.

### Myocellular signaling and mRNA-transcription

Despite WPH being ineffective in augmenting protein synthesis above the PLA trial, WPH elevated phosphorylation in some of the signaling targets involved in translational control of protein synthesis. p-mTOR and p-p70S6K (but not p-eIF4E) showed an elevated relative abundance both immediately and 60 min after completion of 90ss. This confirms the trend shown by Taylor et al., suggesting an enhanced activity of key regulators of protein synthesis during carbohydrate-restricted exercise if a protein feeding stimulus is provided [20]. Still, from our tracer kinetics data it appears that the energy costly process of translation may be relegated. Thus, WPH seems to have induced a more anabolic environment; but the stimulus was not translated into a detectable alteration of the protein synthesis rate. Furthermore, signaling kinases reputed to be key regulators of mitochondrial biogenesis, i.e. p53 and p38MAPK, were unaffected by treatment. The observed development for p-p53 and p-p38MAPK must be interpreted on the basis of the previous evening’s HIIT session and subsequent overnight fast. Presumably, the phosphorylation of these targets was likely already augmented at the time the baseline biopsy. Thus, comparing these results to the growing body of literature showing increases in p53 and p38MAPK phosphorylation as an effect of a single bout of exercise commenced with reduced muscle glycogen availability (for review see Hawley and Morton [3]) would be inappropriate and, indeed, falls beyond the scope of the present study. While the phosphorylation of p53 and p38MAPK was more or less unaffected by exercise, PGC-1α mRNA expression was elevated in both trials. This suggests that signaling cascades preceding transcription of genes encoding proteins involved in mitochondrial biogenesis were activated in response to exercise, and that this positive adaptive response did not appear to be negatively affected by protein feeding prior to exercise.

### Limitations

To isolate the effect of the protein ingestion prior to carbohydrate restricted training, we chose a proof-of-principle approach. We acknowledge that the omission of post-exercise food intake does not directly translate to a real-world setting. Yet, this approach allowed us gain an understanding of the influence of this particular feeding strategy both during and after training. Furthermore, the addition of post-exercise feeding would have posed even greater methodological challenges in regard to the stable isotope tracer techniques. As we measured the fractional synthesis rate only, we were unable to assess the direct effect of WPH on muscle protein breakdown in the working muscle and evaluate the impact on muscle protein net balance, which may have been affected by protein ingestion per se and by the higher level of insulin in WPH compared to PLA.

Measuring protein turnover during feeding and exercise prompts some methodological challenges arising from physiological perturbations. We used plasma tracer enrichments as a surrogate precursor estimate. Under resting conditions, circulating tracer enrichments, are higher than the intramuscular enrichments [24] and the transfer-RNA precursor enrichment [44, 45]. This results in an underestimated FSR. During and immediately after exercise, however, the intramuscular tracer enrichment is approaching the level in the circulation [46], resulting in a slighter overestimation of the real precursor enrichment, hence a reduced underestimation of the FSR. We argue that with the use of precursor estimates in the present study, the FSR at basal and in recovery condition is more underestimated than during exercise. If anything, the difference in FSR during exercise (90ss) compared to the basal and recovery condition is smaller than what we report. Conservatively, we suggest that our quantitative measures should be interpreted as indicative. Still, we find that our collective portfolio of outcomes supports our tracer kinetics data and forms a basis from which a valid conclusion may be drawn. Furthermore, our sample size was small which may have reduced our ability to detect statistical effects for muscle protein turnover. Additionally, it is worth noting, that the participants in the present trial were all men. This potentially limits broader inferences of our findings, i.e. to the female athletic population. Finally, our participants were trained, but not all part of the international elite of endurance athletes. We cannot rule out that training status influence protein turnover at the response to protein supplements.

## Summary

In summary, we provide novel data showing that protein ingested prior to endurance-type exercise in the energy- and carbohydrate-restricted state does not enhance myofibrillar FSR in the working muscles or improve muscle protein balance in the non-working muscles compared to ingestion of non-caloric control. Presumably, this observation is linked to a metabolic priority shift towards energy preservation. During exercise, we observed slight augmentations in muscle protein synthesis rates in the exercising muscles and net balance in the non-exercising muscles, independently of protein feeding. This suggests that the practice of commencing training in a glycogen-depleted/fasted condition to enhance endurance-training adaptations does not appear to pose any significant risk in regard to losing skeletal muscle mass. However, protein breakdown is upregulated following exercise, at least in non-exercising muscles, thus sufficient refueling in terms of protein and carbohydrate is crucial to induce an anabolic hormone response, stimulate glycogen resynthesis and protein synthesis, as well as attenuate protein breakdown.

## Supplementary information

**Additional file 1.** Amino acid profile. Beverage amino acid profile and peptide distribution.

**Additional file 2.** Calculations. Tracer kinetics calculations.

## Data Availability

The datasets used and/or analyzed during the current study are available from the corresponding author on reasonable request.

## Abbreviations

90ss: 90 min steady state cycling
AMPK: AMP-activated kinase
BL: Baseline
BR: Bed rest
COX4I1: Cytochrome c oxidase subunit 4 isoform 1
CPT1B: Carnitine palmitoyltransferase I b
DXA: Dual-energy x-ray absorptiometry
eEF2: Eukaryote elongation factor 2
FFA: Free fatty acid
FSR: Fractional synthesis rates
h: Hour/hours
HIIT: High-intensity interval training
LBM: Lean body mass
mTOR: Mammalian target of rapamycin
p-eIF4E: Phospho-Eukaryotic translation initiation factor 4E
PGC-1α: Peroxisome proliferator-activated receptor gamma coactivator -1α
PLA: Placebo
p-p38MAPK: Phospho-p38 mitogen-activated protein kinases
p-p53: Phospho-p53
p-p70S6K: Phospho-p70S6 kinase
PPO: Peak power output
RPLP0: 60S acidic ribosomal protein P0
SD: Standard deviation
TFAM: Mitochondrial transcription factor A
WPH: Whey protein hydrolysate
