# Supplementary material for: Effects of protein intake prior to carbohydrate-restricted endurance exercise: a randomized crossover trial
Source: J Int Soc Sports Nutr. 2020 Jan 28;17:7. doi: 10.1186/s12970-020-0338-z (PMC6986159; doi:10.1186/s12970-020-0338-z)
Supplement: Supplementary file 1 — Additional file 1. Amino acid profile. Beverage amino acid profile and peptide distribution. [file 12970_2020_338_MOESM1_ESM.pdf]

Additional file 1 – Amino acid profile

**Amino acid (AA)**	**g. AA / 100g. protein**
Alanine	5.2
Arginine	2.5
Aspartic acid	12.6
Cysteine	2.5
Glutamic acid	21.5
Glycine	1.9
Histidine	2.2
Isoleucine	6.4
Leucine	9.3
Lysine	10.9
Methionine	2.0
Phenylalanine	2.4
Proline	7.2
Serine	6.2
Threonine	8.2
Tryptophan	1.0
Tyrosine	2.3
Valine	6.0

Peptide distribution

Mw (Dalton)	Weight %
< 750*	~ 58
750-1250	~ 24
1250-2500	~ 15
>2500	~ 3

*Small peptides (containing 1-6 AA)
