# Supplementary material for: Effects of protein intake prior to carbohydrate-restricted endurance exercise: a randomized crossover trial
Source: J Int Soc Sports Nutr. 2020 Jan 28;17:7. doi: 10.1186/s12970-020-0338-z (PMC6986159; doi:10.1186/s12970-020-0338-z)
Supplement: Supplementary file 2 — Additional file 2. Calculations. Tracer kinetics calculations. [file 12970_2020_338_MOESM2_ESM.pdf]

Additional file 2 - Calculations

Calculations. The phenylalanine kinetics were calculated as described previously [31]. Briefly, net phenylalanine balance was calculated as

$$NB_T = [CU_A + (CU_A \times TTR_A)] - [CU_V + (CU_V \times TTR_V)] \times PF$$

Total flux synthesis was calculated as

$$FT_S = (CU_A \times TTR_A - CU_V \times TTR_V) \times \left(\frac{1 + TTR_A}{TTR_A}\right) \times PF$$

Total flux breakdown was calculated as

$$FT_B = FT_S - [CU_A \times (1 + TTR_A) - CU_V \times (1 + TTR_V)] \times PF$$

where NB_T is the total net phenylalanine balance, CU_A is the arterial concentration of unlabeled phenylalanine, CU_V is the venous concentration of unlabeled phenylalanine, TTR_A is the arterial enrichment of the L-[ring-$^{13}C_6$] phenylalanine [expressed in tracer:tracee ratio (TTR)], TTR_V is the venous enrichment of the L-[ring-$^{13}C_6$] phenylalanine, FT_S is flux total synthesis, FT_B is flux total breakdown and PF is plasma flow.

Vastus lateralis myofibrillar fractional synthetic rate (FSR) was calculated using the standard precourser equation:

$$FSR = \frac{\Delta E_{protein}}{E_{precursor} \times \Delta time} \times 100\%$$

Where $\Delta E_{protein}$ is the difference in tracer enrichment in the myofibrillar protein fraction between two timepoints (hours), $E_{precursor}$ is the weighted mean of the tracer enrichment in the arterial plasma. The conversion of δ-values from the IRMS analyses to TTR was performed using the following formula

$$TTR = 0.0112372 \times \left(\frac{\delta}{1000} + 1\right) \times 100\%$$
